# The *Sw-5* Gene Cluster: Tomato Breeding and Research Toward Orthotospovirus Disease Control

**DOI:** 10.3389/fpls.2018.01055

**Published:** 2018-07-19

**Authors:** Athos S. de Oliveira, Leonardo S. Boiteux, Richard Kormelink, Renato O. Resende

**Affiliations:** ^1^Department of Cell Biology, Institute of Biological Sciences, University of Brasília, Brasília, Brazil; ^2^National Center for Vegetable Crops Research (CNPH), Embrapa Vegetables, Brasília, Brazil; ^3^Laboratory of Virology, Wageningen University and Research Center, Wageningen, Netherlands

**Keywords:** tomato, orthotospovirus, resistance, Sw-5, Sw-5b, NLR, NB-LRR

## Abstract

The *Sw-5* gene cluster encodes protein receptors that are potentially able to recognize microbial products and activate signaling pathways that lead to plant cell immunity. Although there are several *Sw-5* homologs in the tomato genome, only one of them, named *Sw-5b*, has been extensively studied due to its functionality against a wide range of (thrips-transmitted) orthotospoviruses. The *Sw-5b* gene is a dominant resistance gene originally from a wild Peruvian tomato that has been used in tomato breeding programs aiming to develop cultivars with resistance to these viruses. Here, we provide an overview starting from the first reports of Sw-5 resistance, positional cloning and the sequencing of the *Sw-5* gene cluster from resistant tomatoes and the validation of Sw-5b as the functional protein that triggers resistance against orthotospoviruses. Moreover, molecular details of this plant–virus interaction are also described, especially concerning the roles of Sw-5b domains in the sensing of orthotospoviruses and in the signaling cascade leading to resistance and hypersensitive response.

## Introduction

Orthotospoviruses (family *Tospoviridae*) cause substantial losses in crop production worldwide (Pappu et al., 2009). They can be transmitted mechanically, mostly associated with experimental transmission, and by a limited number of thrips species (order *Thysanoptera*) in nature, replicating in both plant and invertebrate hosts (Rotenberg et al., 2015). Until recently, the orthotospoviruses used to be taxonomically classified within the family *Bunyaviridae* (currently reclassified into the new order *Bunyavirales*) together with vertebrate-infecting viruses due to their similar virion shape, genomic organization and, of course, phylogenetic relationship (Adams et al., 2017).

Due to their ability to infect many crops, farmers, seed industry, and researchers have been constantly looking for natural resistance sources against orthotospoviruses. Among few that have been explored commercially (de Ronde et al., 2014), the one from tomato (*Solanum lycopersicum* L.) is the subject of this mini-review. Initially, this resistance source used to be referred to as Sw-5, being utilized in tomato breeding programs. Sequencing of the resistance gene locus, originally from *Solanum peruvianum* Mill. (a wild Peruvian tomato), revealed five paralogs, the so-called *Sw-5* gene cluster (Spassova et al., 2001). Isolation of two resistance gene candidates (RGCs) and their subsequent transformation into tobacco plants helped to demonstrate that the gene copy *Sw-5b* is solely responsible for a broad-spectrum resistance to orthotospoviruses (Spassova et al., 2001; Hallwass et al., 2014; Leastro et al., 2017).

The first sections of this mini-review will describe historical aspects of Sw-5 resistance, from *S. peruvianum* to the first bred commercial tomatoes, genetic characterization of the *Sw-5* gene cluster and the identification of *Sw-5b* as the functional gene copy against orthotospoviruses. The last sections will focus on the identification of the avirulence-determinant/effector from orthotospoviruses and the interplay with and among domains of encoded proteins by different *Sw-5* alleles. The mini-review finishes with some perspectives on challenging research questions for the future that may advance our understanding of the Sw-5b-mediated resistance.

## Sw-5 Resistance: From Peru to a Worldwide Scope

*Solanum peruvianum* varieties naturally occur in the coastal and medium elevated regions of central and southern Peru and in northern Chile (Nakazato et al., 2012). These plants have been targeted as a wild source for crop improvement because of their genetic resemblance to cultivated tomatoes (Nesbitt and Tanksley, 2002). The first reports of *S. peruvianum* harboring a broad resistance source against tomato spotted wilt virus (TSWV) date from the end of the 1930s (Stevens et al., 1991). Since then, efforts have been made to cross this wild tomato with commercial cultivars. One of the first tomato fresh market cultivars originated from such cross was named *Stevens* (Stevens et al., 1991). Subsequent inheritance studies indicated that a single, dominant gene/locus (named as *Sw*-5) was responsible for resistance that was initially found to be effective against a wide array of orthotospovirus isolates from United States (Stevens et al., 1991) and Brazil (Boiteux and de Giordano, 1993).

## Discovery of the *Sw-5* Gene Cluster

In spite of being widely used in tomato breeding programs, the genetic identity of the Sw-5 resistance remained unknown for decades. However, by gene mapping, the resistance locus was first located on the long arm of chromosome 9 of tomato cultivar *Stevens* (Chagué et al., 1996). Two other studies mapped the Sw-5 locus near markers CT220 and SCAR421 (Stevens et al., 1995; Brommonschenkel and Tanksley, 1997) and allowed Folkertsma et al. (1999) to physically fine map the locus and identify Sw-5 RGCs using a bacterial artificial chromosome (BAC) library made from tomato cultivar *Stevens*.

Sequencing of BAC clones containing genomic DNA fragments of the cultivar “*Stevens*” revealed five paralogs (Folkertsma et al., 1999; Brommonschenkel et al., 2000; Spassova et al., 2001). These genes, named *Sw-5a* to *Sw-5e*, encode proteins that contain nucleotide-binding (NB) and leucine-rich repeat (LRR) domains, which are often observed in other plant resistance proteins (van der Biezen and Jones, 1998; Andolfo et al., 2014). From those genes, two highly homologous genes, named *Sw-5a* and *Sw-5b*, mapped close to the markers and exhibited significant resemblance to the tomato nematode and aphid resistance gene *Mi* (Brommonschenkel et al., 2000; Spassova et al., 2001). Furthermore, the presence of a prominent matrix attachment region (MAR) between those two genes (Spassova et al., 2001), and indicative of genomic regions accessed by the nuclear transcription machinery (Stief et al., 1989), supported their RGC signature. To find out which one of those two conferred resistance to TSWV, copies of *Sw-5a* and *Sw-5b* together with their own regulatory sequences were transferred to *Nicotiana tabacum* L. (Spassova et al., 2001). Only transgenic plants transformed with the *Sw-5b* gene were resistant after challenge with TSWV isolates (Spassova et al., 2001), even though the *Sw-5a* and *Sw-5b* genes share a high sequence identity of 97.7% (Spassova et al., 2001; De Oliveira et al., 2016).

In tomatoes carrying Sw-5, high levels of resistance have been observed to TSWV, tomato chlorotic spot virus (TCSV), groundnut ringspot virus (GRSV), and chrysanthemum stem necrosis virus (CSNV) (Boiteux and de Giordano, 1993; Dianese et al., 2011). This broad spectrum resistance is quite unique for a dominant NB-LRR type of resistance gene and contrasts the *Tsw* resistance gene from pepper (*Capsicum chinense* L.) which provides resistance to TSWV isolates only (Boiteux and Deavila, 1994). More recently, *N. benthamiana* plants transformed with the *Sw-5b* gene copy were challenged with six different orthotospoviruses. Besides the aforementioned four orthotospoviruses, *Sw-5b* provided resistance to alstroemeria necrotic streak virus (ANSV) and impatiens necrotic spot virus (INSV) as well (Leastro et al., 2017). Thus, the *Sw-5b* gene alone is sufficient for broad-spectrum resistance, although this applies to phylogenetically related orthotospoviruses that are clustered in the same evolutionary clade (de Oliveira et al., 2012; Lima et al., 2016). Conversely, viruses phylogenetically distant from this “TSWV clade,” e.g., melon yellow spot virus (MYSV), are not sensed and overcome the resistance when mechanically inoculated onto these *Sw-5b* gene-transformed *N-benthamiana* plants (Hallwass et al., 2014).

## Hypersensitive Cell Death Response Triggered by Sw-5b

Another observation made regarding the transgenic tobacco plants involves the activation of a hypersensitive response (HR). This phenotype is usually observed as local necrotic lesions/spots and often appears (within several days) on leaves upon activation of immune protein receptors containing the NB and LRR domains (de Ronde et al., 2014). During HR, infected and neighboring cells are thought to kill themselves to halt the pathogen spread throughout the plant tissue. While the *Sw-5b*-transformed *N. tabacum* plants do not show any HR after mechanical inoculation with TSWV (Spassova et al., 2001), and strengthen the concept that the HR is not the resistance mechanism of NB-LRR proteins (Liu et al., 2010), the *Sw-5b* gene-transformed *N. benthamiana* plants show a clear, concomitant HR (Hallwass et al., 2014; Leastro et al., 2017). The main difference between these two transgenic plants is that in *N. tabacum* expression of the *Sw-5b* gene is governed by its own regulatory sequences (Spassova et al., 2001) while in *N. benthamiana* the *Sw-5b* gene is driven by constitutive expression from the strong 35S promoter of cauliflower mosaic virus (Hallwass et al., 2014). Whether this is the cause for HR induction remains speculative, but recent studies have shown auto-activation of *Sw-5b* when expressed from a highly translatable expression vector (Sainsbury et al., 2009) or in the additional presence of an RNA silencing suppressor (RSS) (De Oliveira et al., 2016).

## Identification of the Avr-Determinant/Effector of Sw-5b

The NB-LRR proteins are also known as NLR proteins, an acronym that also stands to nucleotide-binding (NB) and LRR domains (L)-containing (R) receptors (Wilmanski et al., 2008). Many classes of such proteins have been described in both plants and animals, diverging in structure and activation mode (Ting et al., 2008; Wilmanski et al., 2008; Qi and Innes, 2013). They recognize pathogens by sensing microbial products, usually peptides/proteins (Wilmanski et al., 2008). In plants, these products are commonly referred to as avirulence (avr)-determinants or effectors (de Ronde et al., 2014). “Avirulence” since, once identified, the pathogens cannot cause infection, not being virulent (able to induce disease symptoms).

In order to identify the avr-determinant of Sw-5b, indirect approaches have been performed due to the lack of an orthotospovirus infectious clone (Hoffmann et al., 2001; Lopez et al., 2011; Hallwass et al., 2014; Peiro et al., 2014). The bottleneck in building an infectious clone resides in the fact that the orthotospovirus genetic RNA elements are of negative (non-messenger sense) polarity. Orthotospoviruses have a tripartite single-stranded RNA genome, with each segment named according to its length as Small (S), Medium (M), and Large (L). Whereas the L segment has a negative polarity, the other two present an ambisense genetic organization (King et al., 2012). In total, the orthotospoviruses express six mature proteins: (i) Nucleocapsid (N) and a non-structural protein (NSs) encoded on opposite strands of the S segment; (ii) envelope glycoproteins Gn and Gc, and a non-structural protein (NSm) encoded on opposite strands of the M segment; (iii) and L (an RNA-dependent RNA polymerase) encoded on the viral complementary strand of the L segment. While NSs protein suppresses the RNA silencing machinery (Takeda et al., 2002), NSm protein functions as a cell-to-cell movement protein in plant hosts (Kormelink et al., 1994; Storms et al., 1995; Takeda et al., 2002).

First pieces of evidence already indicated that the avr-determinant of Sw-5b-mediated resistance was encoded in the M segment of TSWV. Reassorted viruses containing the S and L segments of a resistance-inducing (RI) TSWV isolate and the M segment of an RB TSWV isolate overcame the Sw-5 resistance in tomato plants (Hoffmann et al., 2001). Furthermore, comparison of genomes from several RI and RB TSWV isolates pointed toward two amino acid substitutions (C118Y or T120N) in the NSm protein that were associated with the emergence of RB isolates (Lopez et al., 2011). Final evidence for the identification of the avr-determinant came from transient expression analysis of TSWV proteins from binary clones using agrobacteria, in which only NSm triggered HR in plants harboring a functional *Sw-5b* gene copy (Hallwass et al., 2014; Peiro et al., 2014). Moreover, expression of NSm proteins from CSNV and TCSV isolates also triggered HR in those plants, being abrogated by the introduction of mutations C118Y or T120N (Leastro et al., 2017). All these experiments validated NSm as the avr-determinant of Sw-5b.

## Sw-5b Activation Mode

Although there are other variations, most plant NLRs have at their amino (N)-terminus either a Toll and interleukin-1 receptor (TIR) domain or a coiled-coil (CC) domain, which is then followed by the central NB- and carboxy (C)-terminal LRR domains (Meyers et al., 2003). Therefore, NLRs are often also classified as TNLs (TIR-containing NLRs) or CNLs (CC-containing NLRs). The Sw-5 proteins belong to the class of CNLs but are distinct by the presence of an additional extension at their N-terminus, the so-called Solanaceae domain (SD) (De Oliveira et al., 2016). This extra domain has been reported in other Solanaceae CNLs as well (Mucyn et al., 2006). Dissection of Sw-5b has designated important functions to each domain. Using this approach, truncated versions of Sw-5b, containing one or more domains, have usually been expressed by agroinfiltration in wild-type *N. benthamiana* leaves (Chen et al., 2016; De Oliveira et al., 2016).

The Sw-5b protein has a nucleocytoplasmic distribution, with the SD and CC domains (covalently bound) responsible to deliver the protein to the cell nucleus (De Oliveira et al., 2016). This nuclear localization is possibly required for the signal transduction that leads to the resistance mechanism itself. The absence of SD-CC does not interfere with NSm recognition and HR triggering, but transgenic plants transformed with a truncated *Sw-5b* gene lacking these domains are not resistant to orthotospoviruses (Chen et al., 2016). Altogether, these results indicate that resistance and HR are uncoupled events (Chen et al., 2016; De Oliveira et al., 2016). The SD and CC domains are also involved in the activation control of Sw-5b. In the absence of NSm, CC suppresses the HR triggered by NB while SD enhances this inhibition independently (Chen et al., 2016). Interestingly, if NSm is present, SD acts as a positive regulator, relieving the inhibitory effect over NB (Chen et al., 2016).

The NB domain, which also includes the subdomains ARC 1 and ARC 2, is solely responsible for the signaling pathway that leads to HR triggering (Chen et al., 2016; De Oliveira et al., 2016). This domain switches between “off” and “on” states according to its binding to adenosine diphosphate (ADP) or adenosine triphosphate (ATP), respectively (Williams et al., 2011). Overexpression of NB in the presence of RSSs results in HR as observed for the full Sw-5b protein (De Oliveira et al., 2016). Expression of NB alone can also result in a negligible HR that is inhibited in the presence of LRR (NB-LRR covalently bound) (Chen et al., 2016).

**FIGURE 1:**
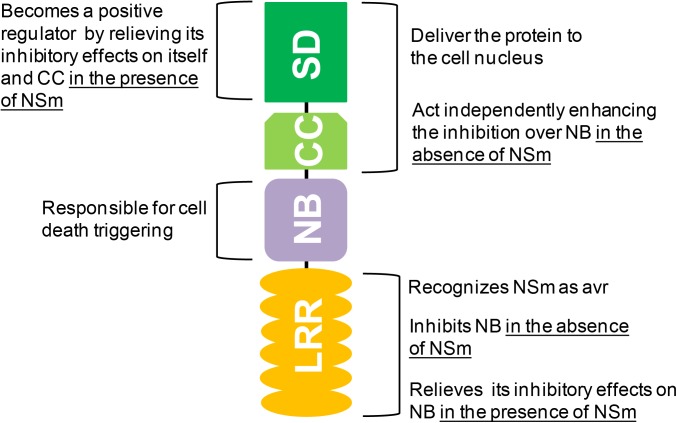
Overview of the (modulating) roles of the amino-terminal Solanaceae (SD) and coiled-coil (CC), central nucleotide-binding (NB), and carboxy-terminal leucine-rich repeat (LRR) domains in Sw-5b activation. Detailed information is found in the manuscript text.

As mentioned before, co-expression of NB-LRR (covalently bound) and NSm triggers a strong HR. If only NB is co-expressed with NSm, no HR is seen (De Oliveira et al., 2016). Thus, LRR recognizes NSm as avr-determinant and activates NB. For most studied plant NLRs, the pathogen is perceived by an indirect interaction between the LRR and the avr-determinant (Dangl and Jones, 2001; van der Hoorn and Kamoun, 2008). Using co-immunoprecipitation assays, Sw-5b NB-LRR domains and NSm were shown to directly interact *in vitro* and *in planta* (Zhu et al., 2017). A conserved 21 amino acid epitope from NSm was sufficient for the interaction and activation of Sw-5b (Zhu et al., 2017). Within the LRR domain, four polymorphic sites were critical for recognition of this NSm epitope (Zhu et al., 2017). As such, Sw-5b is one of the few CNL proteins able to perceive the avr-determinant via direct interaction. **Figure 1** summarizes the functions mapped to Sw-5b domains.

## Tomato Paralogs and Orthologs of *Sw-5b*

In contrast to the findings with Sw-5b, co-expression of Sw-5a and TSWV NSm does not result in HR triggering (De Oliveira et al., 2016), which could explain why *Sw-5a*-transformed tobacco plants are susceptible to TSWV (Spassova et al., 2001). Dissection analysis of Sw-5a, in analogy to Sw-5b, showed that overexpression of the entire Sw-5a protein or its NB domain only leads to auto-activation (HR triggering in the absence of an avr-determinant) (De Oliveira et al., 2016). Considering that *Sw-5a* is the highest conserved paralog of *Sw-5b* (95.1% aa identity), it is likely that some point mutations within the LRR domain disrupted the avr-determinant sensing of Sw-5a. Proof for this could come from testing a chimeric Sw-5a NB-LRR construct, in which its LRR domain has been swapped for the functional one from Sw-5b. So far, no studies have yet been performed with the proteins Sw-5c, Sw-5d, and Sw-5e.

In contrast to *Sw-5a* and *Sw-5b*, the protein product of the most conserved ortholog from susceptible tomato isolines (not bred with *S. peruvianum*), named Sw-5a^S^, is not able to trigger auto-HR at all, even though these three proteins share about 94% aa identity (De Oliveira et al., 2016). Analysis of the Sw-5a^S^ NB domain revealed that a point mutation (Q599R) abolished the ability for HR-induction. A reversion of this single amino acid into the one found in the NB domains of Sw-5a and Sw-5b, restored the ability of triggering HR by the Sw-5a^S^ NB domain (De Oliveira et al., 2016).

**FIGURE 2:**
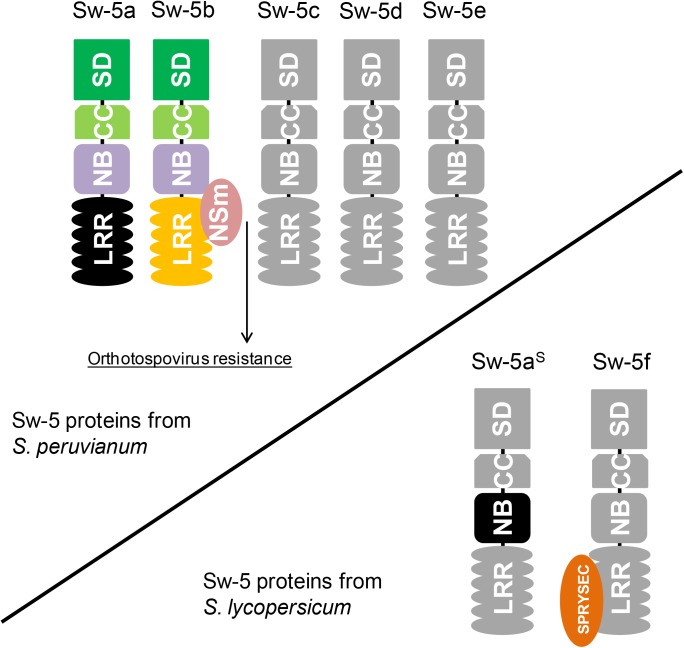
Topology of Sw-5 proteins encoded by paralogs from the *Sw-5* gene cluster and orthologs collected from tomatoes susceptible to orthotospoviruses. The upper schematic encompasses the Sw-5 proteins of *S. peruvianum*. So far, there are only functional studies on Sw-5a and Sw-5b. The latter directly interacts with NSm (orthotospovirus cell-to-cell movement protein), which activates orthotospovirus resistance. On the contrary, Sw-5a is unable to recognize NSm as avirulence (avr)-determinant. The lower schematic shows two Sw-5 proteins found in tomato cultivars susceptible to orthotospoviruses. The most conserved homolog of Sw-5a and Sw-5b, named Sw-5a^S^, contains a mutation in its nucleotide-binding (NB) domain that halts hypersensitive response (HR) triggering. Apart from this, Sw-5a^S^ cannot recognize NSm as avr-determinant. Sw-5f has been first reported interacting with an effector (SPRYSEC-19) of nematode *Globodera rostochiensis* that inhibits HR triggered by several resistance proteins.

Susceptible and resistant tomato plants can be easily differentiated by using a co-dominant molecular marker that detects a series of conserved deletions (of variable sizes depending upon the cultivar) upstream of the coding sequence of *Sw-5a^S^* (Dianese et al., 2010). Apart from these deletions, the regulatory sequences of *Sw-5a*, *Sw-5b*, and *Sw-5a^S^* are highly conserved. With the availability of the tomato genome sequence (Sato et al., 2012), all *Sw-5b* orthologs from the susceptible tomato cultivar “*Heinz 1706*” could be mapped. Including *Sw-5a^S^*, three complete *Sw-5* genes (containing the SD-CC-NB-LRR domains) and a truncated gene are found in chromosome 9 of tomato cultivar “*Heinz 1706*” (Turina et al., 2016). One of these *Sw-5* genes encodes a protein referred to as Sw-5f, although it is an ortholog and not a paralog of Sw-5b. The Sw-5f protein interacts with the effector protein SPRYSEC-19 from nematode *Globodera rostochiensis* (Rehman et al., 2009). This effector inhibits HR triggered by many CNL proteins, including an auto-active mutant of Sw-5b (Postma et al., 2012). **Figure 2** schematizes some Sw-5-related proteins found in resistant and susceptible tomatoes to orthotospoviruses.

## Conclusions and Perspectives

Few plant immune receptors against viruses have been deeply studied to date. Apart from tomato Sw-5b, most of the efforts concentrated on the resistance mechanisms orchestrated by potato Rx against potato virus X (PVX) and tobacco N against tobacco mosaic virus (TMV) (de Ronde et al., 2014). The findings obtained from studying these proteins, especially concerning their activation mode, can be extrapolated to other plant NLR proteins with a similar topology (domain organization). Since HR is not the resistance mechanism itself, the actual downstream signaling cascade and cellular events leading to Sw-5b-mediated resistance remain to be elucidated. In this context, identifying plant proteins that directly or indirectly interact with Sw-5b as well as identifying the genes that are up- or down-regulated during the resistance response may provide further clues for this.

We do not rule out that in addition to Sw-5b, the other Sw-5-related proteins trigger resistance to different pathogens. This has been observed for NLRs from potato, in which the paralogs Rx and GPa2 trigger resistance to PVX and nematode *G. pallida*, respectively (van der Vossen et al., 2000). Taking into consideration that *Sw-5a* and *Sw-5a^S^* share high identity with *Sw-5b*, these two homologs could still be reverted to functional genes against orthotospoviruses. However, as we discussed in previous sections, point mutations in single domains, e.g., only in LRR that recognizes NSm as avr-determinant, may not be sufficient. Structural and modeling studies need to be performed to identify (a combination of) modifications that have to be implemented to turn non-functional copies into one that resembles the functional Sw-5b. The insertion of these modifications in the tomato genome, especially in those containing only *Sw-5a^S^*, could be done by genome editing tools such as CRISPR and TALENs (Gaj et al., 2013).

## Author Contributions

AdO organized and wrote most of this manuscript. LB, RK, and RR revised and added complementary information.

## Conflict of Interest Statement

The authors declare that the research was conducted in the absence of any commercial or financial relationships that could be construed as a potential conflict of interest.
